# Specialized Nursing-Led Interventions for Bladder Cancer Management: A Scoping Review of Evidence and Clinical Outcomes

**DOI:** 10.3390/medicina62010185

**Published:** 2026-01-16

**Authors:** Omar Alqaisi, Patricia Tai, Guy Storme

**Affiliations:** 1 Nursing Department, Al-Zaytoonah University, Airport Street, Amman P.O. Box 130, Jordan; 2 Department of Oncology, University of Saskatchewan, 105 Administration Place, Saskatoon, SK S7N 5A2, Canada; pat961@usask.ca; 3 Department Radiation Oncology, UZ Brussel, Laarbeeklaan 101, 1090 Jette, Belgium; guy.storme@telenet.be

**Keywords:** bladder cancer, nursing interventions, quality of life, patient satisfaction, psychosocial support

## Abstract

*Background and Objectives*: Bladder cancer (BC) represents a significant global health burden, ranking as the tenth most commonly diagnosed malignancy worldwide, with an incidence rate of 5.6 per 100,000 person-years annually. The research team aimed to summarize evidence on specialized nursing-led interventions for bladder cancer management across the disease continuum. *Materials and Methods*: This scoping review used the Preferred Reporting Items for Systematic Reviews and Meta-Analyses extension for Scoping Reviews (PRISMA-ScR) methodology to search four databases from January 2018 to November 2025. *Results*: This concise but informative scoping review of 20 studies revealed substantial clinical and patient-reported benefits from specialized nursing interventions. Enhanced recovery after surgery (ERAS) protocols incorporating structured nursing care demonstrated a 35% reduction in postoperative complications. Integrated nursing interventions during postoperative intravesical therapy significantly improved patient satisfaction, treatment compliance, and self-efficacy while reducing anxiety and depression. Digital health platforms, including internet-based and mobile applications, proved effective in reducing caregiver burden, enhancing disease knowledge, and improving coping strategies. Preoperative stoma education and postoperative ostomy care management significantly improved self-efficacy, stoma care knowledge, and overall health-related quality of life. Psychosocial interventions, including cognitive behavioral therapy and mindfulness-based approaches, demonstrated significant improvements in quality of life and reductions in fear of recurrence, depression, and anxiety. However, a critical evidence gap exists regarding bladder cancer-specific mental health interventions. *Conclusions*: Specialized nursing-led care plays a critical role in strengthening clinical and assistive practice in bladder cancer. Evidence from this scoping review shows that nursing-led interventions significantly improve clinical outcomes, patient satisfaction, symptom management, and quality of life across all phases of bladder cancer care while reducing caregiver burden and enhancing psychological well-being for both patients and families, reinforcing the value of integrating specialized nursing roles into routine bladder cancer management.

## 1. Introduction

Bladder cancer (BC) represents a significant global health burden, ranking as the tenth most commonly diagnosed malignancy worldwide [1]. In 2020, approximately 573,000 new cases and 213,000 deaths were reported globally, with age-standardized incidence and mortality rates of 5.6 and 1.9 per 100,000 person-years, respectively [1]. Projections indicate a substantial increase in disease burden, with a 73% rise in annual cases and 87% increase in deaths by 2040, primarily driven by population growth and aging demographics [1].

The cancer exhibits marked sex disparities, with incidence and mortality rates approximately four-fold higher in men than women [2]. Geographically, the highest incidence rates are observed in Southern and Western European populations [2,3], while mortality rates peak in the Northern Africa region [3]. In Asia due to smoking, the bladder incidence is also increasing [4,5]. Looking into carcinogens and interaction with urinary microbiomes (*urobiomes*) leading to urothelial cancer [6], *Fusobacterium*, *Streptococcus*, *Veillonella*, and *Actinomyces* were more frequently found in bladder cancer patients compared to normal people [7,8]. Possible contributing factors are chronic inflammation, immune modulation, and increased production of nitrosamines (a carcinogen) [9]. These urobiomes also affect response to chemotherapy [10]. Lastly, pesticides have been correlated with carcinogenesis and development of chemoresistance [11].

Beyond its epidemiological significance, bladder cancer imposes considerable economic and humanistic burdens on healthcare systems and patients [12]. It is characterized by high recurrence rates, ranging from 30% to 54% for local recurrence and up to 50% for distant metastases following radical cystectomy, necessitating lifelong surveillance and repeated interventions [13]. The disease carries the highest lifetime treatment costs per patient among all cancers, with healthcare expenditures exceeding USD 6.5 billion annually in the United States alone [14] with almost a meager increase in relative survival of about 2–3% over 20 years despite new diagnostic tools [14]. Patients experience substantial symptom burden including hematuria, lower urinary tract symptoms, pain, and psychological distress, which profoundly impact health-related quality of life (HRQoL) across the disease trajectory [15,16]. For those undergoing radical cystectomy with urinary diversion, challenges extend to ostomy management, body image concerns, sexual dysfunction, and significant adaptation to altered physiological function [17].

The multifaceted nature of bladder cancer care necessitates comprehensive management strategies that extend beyond conventional medical and surgical treatments. Nursing interventions have emerged as critical components in optimizing patients’ outcomes throughout the cancer continuum—from diagnosis and treatment through survivorship and palliative care [18,19]. Evidence increasingly demonstrates that nursing-led care significantly improves multiple dimensions of patient outcomes [20]. Systematic reviews and meta-analyses have shown that nursing interventions reduce chemotherapy-related complications, decrease postoperative infection rates [20,21], improve wound healing, enhance treatment adherence, and optimize symptom management. Enhanced recovery after surgery (ERAS) protocol incorporating structured nursing has demonstrated reduced hospital length of stay, lower complication rates, and improved functional recovery/quality of life following radical cystectomy [22]. Specialized nursing interventions of ostomy care, monitoring have been associated with improved quality of life, enhanced self-efficacy, reduced anxiety and depression, and increased patient satisfaction [2,23,24]. Similar integrated approaches are evident in colorectal cancer care, where nurse-led interventions enhance screening rates, reduce treatment-related complications, and improve patient quality of life through education, genetic counseling, and palliative support across primary, secondary, and tertiary prevention levels [25,26]. The latter publication covered different nursing roles after reviewing 117 studies: “At the primary level, the most important role related to educating people to prevent cancer and reduce risk factors. At the secondary level, the roles consisted of genetic counseling, stool testing, sigmoidoscopy and colonoscopy, biopsy and screening test follow-ups, and chemotherapy intervention, while at the tertiary level, their roles were made up of pre and postoperative care to prevent further complications, rehabilitation, and palliative care. Nurses at various levels of prevention care also act as educators, coordinators, performers of screening tests, follow-up, and provision of palliative and end-of-life care” [26].

More examples of a complete modern and integrated clinical and assistive practice perspective are: advanced educational programs of breast cancer nurses [27], nurse-led programs to facilitate enrollment to pediatric oncology [28], and teaching oncology nurses a psychosocial intervention for advanced cancer [29].

Despite this growing body of evidence, significant gaps persist in the literature regarding nursing-led interventions for bladder cancer management [16,30]. Current research remains fragmented, with limited synthesis of evidence across the diverse spectrum of nursing interventions spanning perioperative care, symptom management, psychosocial support, patient education, survivorship care and palliative services. Standardized protocols and evidence-based guidelines specifically tailored to nursing practice in bladder cancer care are lacking in many healthcare settings [31]. The optimal models for nurse-led care delivery, including the roles of clinical nurse specialists, advanced practice nurses, and nurse navigators, remain incompletely defined. Furthermore, there is insufficient understanding of how nursing interventions can be integrated within multidisciplinary team frameworks to maximize effectiveness and cost-efficiency [31]. Research gaps also exist regarding nursing interventions for underserved populations, long-term survivorship needs, and the implementation of innovative care delivery models such as telehealth and digital health platforms for continuous nursing support [32]. Therefore, we synthesized the current evidence on nursing-led interventions in bladder cancer management, evaluated their impact on clinical outcomes and quality of life, identified best practices and effective care models, and delineated knowledge gaps that warrant future investigation.

## 2. Materials and Methods

### 2.1. Study Design and Objectives

This study was conducted as a scoping review to systematically map the evidence regarding nursing-led interventions for bladder cancer. The methodology followed the framework developed by Arksey and O’Malley [33]. The report adheres to the Preferred Reporting Items for Systematic Reviews and Meta-Analyses extension for Scoping Reviews (PRISMA-ScR) guidelines [34].

The primary objective was to synthesize evidence on specialized nursing interventions across the bladder cancer care continuum. By consolidating the available evidence, the secondary objective is to provide evidence-based guidance for oncology nurses, inform the development of standardized nursing protocols and support multidisciplinary care optimization, with an ultimate goal to improve patient-centered outcomes across the bladder cancer care continuum.

This review was guided by the following research question: “What is the nature and effectiveness of nursing-led interventions on clinical and patient-reported outcomes in bladder cancer management?”

### 2.2. Search Strategy

A comprehensive search was conducted across four electronic databases: PubMed, Scopus, ScienceDirect, and CINAHL, covering the period from January 2018 to November 2025. The search strategy was developed iteratively by the research team. To ensure full transparency as requested, the checklist and complete search strings for all databases, along with the method used to develop them, are provided in Supplementary Tables S1 and S2.

Search terms combined keywords using Boolean operators including (“nursing interventions” OR “nurse-led” OR “nursing care” OR “nursing management”) AND (“bladder cancer” OR “urothelial carcinoma”) AND (“outcomes” OR” quality of life” OR “patients’ satisfaction” OR “symptoms management”). The final search was conducted on 30 November 2025. Hand-searching of reference lists from all included studies and relevant systematic reviews was performed to identify additional eligible studies. Forward and backward citation tracking was conducted using Google Scholar and Web of Science. PRISMA-ScR does not require study protocols to be prospectively registered.

### 2.3. Eligibility Criteria

Studies were included if they met the following criteria: (1) Focused specifically on nursing-led interventions or nursing management; (2) Involved patients with bladder cancer at any disease stage; (3) Reported clinical outcomes or patient-reported outcomes, e.g., quality of life (QoL), satisfaction; (4) Published in English between 2018 and 2025. Exclusion criteria included studies not specific to bladder cancer, lacking a distinct nursing component, non-peer-reviewed articles, and non-English publications.

As a scoping review, our methodological objective differs from that of a systematic review. According to the PRISMA-ScR (Preferred Reporting Items for Systematic reviews and Meta-Analyses extension for Scoping Reviews) guidelines, scoping reviews aim to map the breadth and nature of existing evidence across heterogeneous study designs, including both primary research and the secondary literature.

### 2.4. Study Selection and Data Extraction

**Figure 1:**
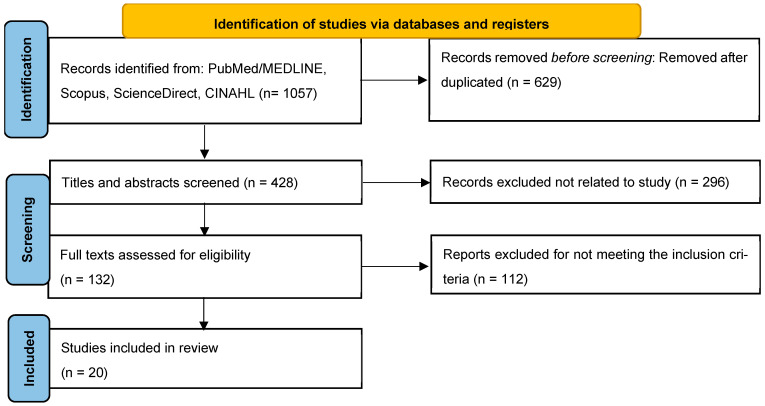
Preferred reporting items for systematic review and meta-analysis extension for Scoping Reviews (PRISMA-ScR) flow diagram of studies to include in this scoping review.

Two independent reviewers (O.A. and P.T.) screened titles/abstracts and later full-text articles. Discrepancies were resolved through consensus. The selection process is detailed in the PRISMA-ScR flow diagram (Figure 1). Data were extracted using a standardized form, capturing the following: Author, Purpose, Settings, Sample size, Study design, and Main findings (Table 1). A completed PRISMA-ScR reporting checklist is provided in Supplementary Table S1 in the Supplementary Materials.

The PRISMA-ScR flow diagram (Figure 1) illustrates the study selection process, resulting in 20 studies included in the final review. In Table 1, data extraction was conducted using a standardized form to capture study characteristics (author, year, country, population, disease stage), nursing intervention types and components, study design and setting, sample size, clinical and patient-reported outcomes (e.g., quality of life, patient satisfaction, anxiety, depression, treatment compliance, caregiver burden, clinical outcomes) and key findings. Consistent with scoping review methodology, no formal risk of bias assessment was conducted. The 20 included studies comprised diverse designs: randomized controlled trials (*n* = 5), systematic reviews and meta-analyses (*n* = 3), comparative studies (*n* = 5), qualitative studies (*n* = 1), clinical guidelines (*n* = 2), scoping reviews (*n* = 2), and other evidence synthesis designs (*n* = 2). Studies were conducted across multiple countries and healthcare settings, including hospital oncology wards, urology departments, tertiary care centers, preoperative clinics, and virtual telehealth platforms. Sample sizes of primary research studies ranged from feasibility pilots (*n* < 20) to large randomized trials (*n* > 300). The extracted data encompassed nursing interventions spanning perioperative care, psychosocial support, stoma education, digital health platforms, symptom management and survivorship care, with outcomes measured across clinical, psychological and patient-reported domains.

### 2.5. Critical Appraisal and Synthesis

Consistent with the PRISMA-ScR guidelines for scoping reviews [34], a formal assessment of methodological quality (risk of bias) of the included studies was not performed. The purpose of this review was to map the breadth of available evidence rather than to assess the quality of individual studies for meta-analysis. Data were synthesized descriptively to categorize interventions and summarize their impact on patient outcomes. We noted that, for cancer patients in general, psychosocial support publication is available, although not specifically for bladder cancer patients [50].

## 3. Results

**Figure 2:**
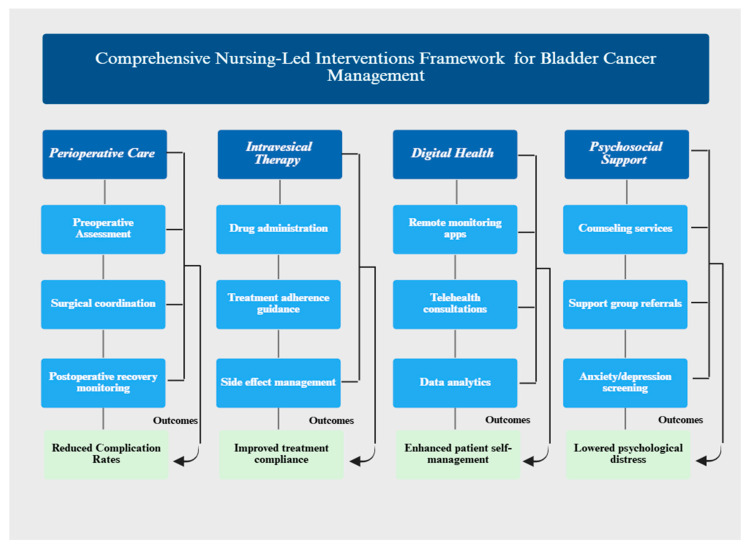
The framework illustrates the intervention components, delivery mechanisms and corresponding clinical and patient-reported outcomes for each category: perioperative care and enhanced recovery after surgery (ERAS) protocols, intravesical therapy management, digital health interventions and psychosocial support services.

Figure 2 presents a comprehensive framework synthesizing the four primary categories of nursing-led interventions for bladder cancer management identified across the 20 included studies.

### 3.1. Perioperative Care and Enhanced Recovery After Surgery (ERAS) Protocols

Nursing-led perioperative interventions demonstrated significant improvements in clinical outcomes following radical cystectomy, indicating substantial clinical benefits. Ashraf reported that integrated ERAS protocols significantly reduced hospital length of stay from 17 to 11 days (35% reduction; *p* < 0.05) and decreased complication rates by 38% compared to traditional care [37]. Similarly, Leminski, A. et al. demonstrated that combined educational and psychological support programs significantly reduced perioperative anxiety and depression in patients undergoing radical cystectomy (*p* < 0.001) [43]. Quality indicators for bladder cancer services emphasized the critical role of nursing interventions in preoperative counseling, stoma site marking, and ERAS protocol implementation [39] (Table 2).

### 3.2. Intravesical Therapy Management: Treatment Compliance and Patient Satisfaction

Wang et al. evaluated integrated nursing interventions during postoperative intravesical for non-muscle invasive bladder cancer (NMIBC) in a randomized controlled trial involving 100 patients [18]. The study demonstrated that comprehensive nursing care significantly improved patient satisfaction scores, treatment compliance, self-efficacy and quality of life while reducing anxiety [18]. Song et al. assessed extended nursing services combined with atezolizumab immunotherapy in 126 bladder cancer patients following endoscopic bladder resection, improving renal function preservation, quality of life, patients’ satisfaction, and significantly reducing caregiver burden, anxiety, and depression (*p* < 0.05) [35] (Table 3).

### 3.3. Digital Health and Telehealth Interventions: Implementation and Outcomes

Digital health platforms emerged as effective modalities for delivering continuous nursing support with demonstrated scalability. In 2022, Fan et al. evaluated internet-based health education for caregivers of stoma patients during the COVID-19 era, finding that the intervention significantly reduced caregiver burden and enhanced coping abilities (*p* < 0.05), with documented improvements in caregiver anxiety and stress management [24].

Kim et al. developed and tested a mobile-based mental health program for non-muscle invasive bladder cancer (NMIBC) patients using the Kakao talk platform (pilot feasibility study), demonstrating potential for improving mental health outcomes [42]. Diefenbach et al. also created a web-based CRIS (cancer recovery information system) platform specifically for bladder cancer survivors, showing high usability and addressing practical, psychosocial, and educational needs post-cystectomy [41]. Zhang et al. implemented continuous nursing interventions via an “internet plus” platform for 43 advanced bladder cancer patients with hematuria, resulting in improved coping styles, enhanced disease knowledge, reduced caregiver burden, and increased patients’ satisfaction [36] (Table 4).

### 3.4. Stoma and Ostomy Care Management: Education and Self-Care Outcomes

Zhang and Qi conducted a systematic review of 10 studies on stoma education and identified that preoperative history was critical for psychological preparation, while postoperative interventions significantly improved self-efficacy and health-related quality of life (HRQoL) in urostomy patients [38]. Wulff-Burchfield et al. evaluated nurse-led preoperative stoma education involving 24 patients and caregivers, demonstrating that interactive education with patient advocates optimally prepared patients for ostomy management and significantly reduced psychological distress [45]. Ding et al. conducted a randomized controlled trial with 340 bladder cancer patients with permanent ostomies, showing that peer-led education significantly improved stoma care knowledge (*p* < 0.001), attitude, practices, and overall quality of life compared to routine nursing [47]. Recently, Zhang and Qi et al. in 2025 performed a narrative review of caregiver burden and nursing education for bladder cancer patients with urinary diversion, concluding that nurse-led stoma education enhanced caregiver comprehension and significantly reduced burden and stress through targeted educational interventions [46] (Table 5).

### 3.5. Psychosocial Support and Mental Health Interventions: Addressing Critical Care Gaps (Table 6)

Bessa et al. conducted a systematic review of supportive mental well-being interventions for bladder cancer patients and identified a critical gap: no bladder cancer-specific mental health interventions were found in the literature [30], although there are publications for all cancer sites in general as noted above. This represents a significant evidence gap that nursing-led programs should address. In 2023, Grassi et al. developed the European Society of Medical Oncology (ESMO) clinical practice guidelines recommending cognitive behavioral therapy and mindfulness-based interventions for managing anxiety and depression in adult cancer patients, noting these conditions are common but under-recognized [40]. In addition, Qian et al. evaluated a gratitude nursing program for fear of cancer recurrence in 80 bladder cancer patients, demonstrating significant improvements in quality of life and significant reduction in fear, depression and anxiety compared to routine care [51]. Peng et al. also assessed a people-oriented nursing model in psychological status in 80 bladder cancer patients, showing reduced anxiety (*p* < 0.05) and depression (*p* < 0.05) and improved quality of life compared to conventional nursing approaches [23]. Thomas et al. conducted a systematic review of 17 studies involving 2572 patients, identifying significant risk factors for psychological distress including advanced disease stage, younger age, female sex, and preoperative anxiety, while social support served as a protective factor [44]. This large study identified specific at-risk populations requiring targeted nursing intervention. In 2025, Alqaisi et al. published two studies on sexual health of cancer patients, and these are relevant to bladder patients since sexual dysfunction is common after surgery or radiotherapy [52,53]. The take-home message for nurses to conquer anxiety about obtaining sexual history is by standardized nursing chronicles, workshops with role-play and more education on sexual health in the nursing curriculum.

**Table 6:** Useful summary studies on mental health of patients with bladder cancer.

Author	Study Type	Population (*n*)	Interventions/Key Findings	Statistical Significance
Bessa [30]	Systematic review	BC patients (literature)	Critical gap identified: BC-specific mental health interventions found	Highlights urgent need for intervention development
Grassi [40]	Clinical practices guidelines	Adult cancer patients	Recommended CBT and mindfulness-based interventions; anxiety/depression underrecognized	Guidelines-level evidence; expert consensus
Qian [51]	Comparative study	80 BC patients	Gratitude nursing program: improve QoL, reduced fear/depression/anxiety	Significant improvement vs. routine care (*p* < 0.05)
Peng [23]	Comparative study	80 BC patients	People-oriented nursing model: reduced anxiety/depression, improved QoL	Anxiety/depression reduction statistically significant (*p* < 0.05)
Thomas [44]	Systematic review of 17 studies	2572 patients	Risk factors identified (advanced stage, younger age, female sex); social support as a protective factor	Meta-analysis of psychological distress outcomes

BC: bladder cancer; CBT: cognitive behavioral therapy; *n*: patient number; and QoL: quality of life.

## 4. Discussion

### 4.1. Clinical Outcomes: Quantifiable Benefits

Specialized nursing-led interventions demonstrated measurable improvements in key clinical parameters with substantial cost-effectiveness implications. Hospital length of stay following radical cystectomy decreased significantly with ERAS protocols, reducing hospitalization from 17 to 11 days (35% reduction) [37], representing potential cost savings of 6 hospital days per patient. The total rate of complications in the intervention group was significantly lower than that in the control group (18.31% vs. 31.13%, *p* < 0.05) [47]. Treatment adherence and chemotherapy completion rates improved substantially as well.

Another important study showed that extended nursing services with systemic therapies preserved renal function in patients receiving atezolizumab immunotherapy, an important outcome for long-term patient survival and quality of life [35]. Intravesical therapy protocols, when coupled with comprehensive nursing support, demonstrated improved patient compliance (100% vs. 84%, *p* < 0.05) and reduced adverse effects.

### 4.2. Patient-Reported Outcomes (PRO): Quality of Life and Psychological Benefits

All included studies reporting quality of life measures demonstrated significant improvements across physical, cognitive, emotional, role, and social function domains following nursing intervention. In fact, multiple studies showed statistically significant reductions in anxiety and depression scores (*p* < 0.05 to *p* < 0.001) with nursing interventions compared to standard care. Wang et al. demonstrated in their randomized controlled trial that physical, role, cognitive, emotional function and social functions were all significantly improved (all *p* < 0.05) [18]. They reported significant reduction in anxiety and depression, with improved patient satisfaction across all intervention types. Preoperative and postoperative nursing education significantly enhanced self-efficacy, particularly in ostomy care management (GSES scores: 35.47 ± 2.31 vs. 31.02 ± 2.27; *p* < 0.05).

Peer-led stoma education improved stoma care knowledge (*p* < 0.001), attitudes, practices, and overall quality of life in 340 bladder cancer patients with permanent ostomies [47]. Structured nursing programs reduced fear of cancer recurrence and improved psychological well-being in bladder cancer survivors [51].

### 4.3. Caregiver-Related Outcomes: Extended Impact Beyond Patients

Multiple studies demonstrated that nursing interventions significantly reduced caregiver burden through comprehensive education, psychosocial support, and digital health platforms. Nurse-led education enhanced caregiver comprehension of stoma care and disease management, leading to improved confidence and reduced stress [46]. Internet-based interventions improved caregiver coping strategies and significantly reduced caregiver burden and anxiety (*p* < 0.05) [24].

Song et al. reported that extended nursing services combined with systemic therapy significantly reduced caregiver burden, anxiety, and depression in relatives of bladder cancer patients (*p* < 0.05) [35]. Digital health platforms addressing caregiver needs demonstrated high effectiveness in supporting family members during the patient’s cancer journey, with specific benefit during the COVID-19 pandemic when in-person support was limited [32].

### 4.4. Effectiveness of Nursing Interventions

One may ask that, in standard care, there are always nurse interventions and so what are the differences from those described here? Our nursing interventions here are defined as specially trained nurses to cope with bladder cancer. The 20 included studies provide evidence that nursing-led interventions significantly improve clinical outcomes, patient-reported outcomes (PRO), and caregiver well-being across the bladder cancer care continuum.

Primary beneficial evidence-based interventions:

- Perioperative ERAS protocols.
- Integrated nursing support during intravesical therapy improves patient satisfaction, treatment compliance and anxiety/depression.
- Extended nursing services with systemic therapy: Renal function preservation; quality of life enhancement; and caregiver burden reduction.
- Digital health platforms: Continuous support delivery; caregiver burden reduction; disease knowledge improvement; feasible implementation during COVID-19 pandemic.
- Comprehensive stoma education: Improved stoma knowledge, self-efficacy and quality of life.
- Structured psychosocial interventions: Improve anxiety, depression and fear of recurrence.

There is another study of advanced nurses playing an important role in the emergency and critical care that corroborates this study [54,55]. With a shortage of physicians in underserved areas, Canada already has nurse practitioners and advanced nurses to improve healthcare access. Therefore, this study has practical value and likely can be generalized to other disciplines other than bladder cancer.

### 4.5. Scientific Rigor and Evidence Synthesis

As a scoping review following PRISMA-ScR guidelines, this review aimed to map evidence breadth rather than provide quantitative effect estimates. The strength of evidence varies by intervention type: ERAS protocols and psychosocial interventions are supported by RCTs and comparative studies (Level I–II evidence), demonstrating consistent benefits including a 35% reduction in complications [37] and significant improvements in quality of life [40,41]. Stoma education programs show strong evidence from RCTs for improved self-efficacy and quality of life [38,43], while digital health platforms represent an emerging area requiring further investigation (Level III evidence) [36,42].

The inclusion of both primary studies and secondary literature (systematic reviews, meta-analyses) enabled comprehensive evidence mapping while accepting that some patient populations may be represented across multiple sources. The heterogeneity in study designs, populations, interventions, and outcomes precluded meta-analysis but allowed identification of consistent patterns across varied settings. Critical evidence gaps include the absence of bladder cancer-specific mental health interventions [30], limited long-term follow-up data, and insufficient implementation of scientific research examining cost-effectiveness and scalability.

### 4.6. Perspectives for Clinical and Assistive Practice in Bladder Cancer Care

In summary, these include early detection, symptom management, and holistic support. Nursing interventions play a vital role in monitoring urinary symptoms, pain, and postoperative infection following procedures such as transurethral resection of the bladder or cystectomy. Nursing responsibilities also include patient education on treatment options, catheter care, lifestyle adjustments, body-image concerns, and self-esteem, as well as providing emotional support for anxiety related to diagnosis and treatment side effects. Assistive strategies such as continence aids, pelvic floor exercises, and rehabilitation programs help improve patient independence, support caregivers, and enhance quality of life. Thus, patient-centered care improves treatment outcomes and greatly supports patients and families throughout their cancer journey.

### 4.7. Evidence Gaps and Future Research Directions

Bessa et al. identified a critical gap in bladder cancer-specific mental health interventions, noting the absence of tailored psychosocial support programs for this patient population [30]. The review revealed that current nursing interventions remain fragmented, with limited integration across the care continuum from diagnosis through survivorship. Additionally, there is insufficient evidence regarding nursing interventions for underserved populations and long-term survivorship needs beyond the immediate treatment period.

Thomas et al. highlighted the need for additional research on psychological distress management, particularly for younger female patients with advanced disease stages [44]. Most included studies (60%) focused on the treatment phase, with limited evidence for supportive nursing interventions during active surveillance and long-term follow-up periods. Furthermore, implementation and scalability of digital health interventions require additional investigation across diverse healthcare settings and patient populations.

Research gaps identified include the following: (a) standardized protocols for bladder cancer-specific mental health nursing interventions; (b) economic analyses comparing cost-effectiveness of nursing interventions versus standard care; (c) long-term survivorship outcomes beyond 2–5 year follow-up; (d) sexual health after treatment [56], across different cultures especially those which regard sex as a taboo topic [52] and (e) comparative effectiveness studies in low-resource and middle-income countries.

### 4.8. Limitations of the Current Study

This scoping review has several limitations. First, consistent with PRISMA-ScR guidelines, no formal risk-of-bias assessment was performed, as the primary objective was to map evidence breadth rather than critically appraise study quality. Second, the inclusion of diverse study designs (RCTs, comparative studies, systematic reviews, and guidelines) alongside primary research precluded meta-analysis but enabled comprehensive evidence mapping. Third, language restrictions to English publications may have excluded relevant studies from non-English-speaking regions with high bladder cancer burden. Fourth, the predominance of studies from high-income countries (United States, Germany, China, Australia, Canada) limits generalizability to low- and middle-income settings where healthcare infrastructure and nursing resources differ substantially. Fifth, most studies reported short-to-medium-term outcomes with limited long-term follow-up data beyond 12 months. Sixth, underrepresentation of specific populations, including elderly patients (≥75 years), racial and ethnic minorities, and those with significant comorbidities, limits applicability of findings. Finally, few studies addressed implementation factors such as cost-effectiveness, resource requirements, or scalability, limiting practical translation into clinical practice. Despite these limitations, this review provides a comprehensive synthesis of evidence supporting specialized nursing interventions across the bladder cancer care continuum while identifying critical knowledge gaps for future research.

## 5. Conclusions

Nurses have an important role to play in patient education in general. Hopefully the message comes across to the readers:

(a) The answer to our research question is that nursing-led interventions on clinical and patient-reported outcomes in bladder cancer management are effective. Specially trained nurses for bladder cancer care are much more efficacious and cost-effective compared with usual care due to reduction in intervention, length of stay in hospitals and complications.
(b) The impact on psychological well-being of caregivers and patients due to education shows the benefit in HRQoL and improved patient satisfaction.
(c) Due to possible prescription of drugs with nephrotoxicity, strict evaluation of renal function can avoid other complications such as dialysis, increasing psychological burden and cost.
(d) Continuous support by telemedicine and other health platforms improve the well-being of patients and caregivers.
(e) Patient education in general and more particularly stoma care improves quality of life as self-sufficient, confident patients. Education of the patient and caregiver and patient results in less psychological harm and better sexual health.
(f) Clinical and assistive care for bladder cancer focuses on early detection, symptom management, and holistic support through nursing interventions such as monitoring symptoms, providing education, and addressing emotional and lifestyle needs. Assistive strategies like continence aids, pelvic floor exercises, and rehabilitation programs further promote independence, enhance quality of life, and strengthen patient-centered care throughout the cancer journey.

## Figures and Tables

**Table 1:** Summary of 20 studies included in this review.

Author	Purpose	Settings	Sample Size	Study Design	Main Findings
Wang [18]	Evaluate effectiveness of integrated nursing intervention on patient outcomes during postoperative intravesical installations for NMIBC	Hosp oncology ward	*n* = 100 NMIBC patients	Comparative RCT	Integrated nursing interventions significantly improved patient satisfaction, treatment compliance, self-efficacy and QoL; reduced anxiety and depression
Song [35]	Assess efficacy of long-term extended nursing services combined with atezolizumab in BC patients after endoscopic bladder resection	Hosp urology department	*n* = 126 BC patients	Randomized controlled trial	Extended nursing services improved renal function, QoL, and satisfaction; reduced caregiver burden, anxiety and depression
Zhang [36]	Evaluate continuous nursing interventions via internet plus platform for advanced BC patients with hematuria	Tertiary hosp	*n* = 43 advanced BC patients	Retrospective observational study	Internet plus nursing improved coping style, disease knowledge, reduced caregiver burden, increased patient satisfaction
Ashraf [37]	Compare integrated ERAS protocol with traditional preoperative care in radial cystectomy	Tertiary referral urology center	*n* = 94 BC patients	Retrospective comparative	ERAS significantly reduced hosp stay (11 vs. 17 days, *p* < 0.0001), faster recovery, reduced complications by 38%
Bessa [30]	Systematic review of supportive mental well-being intervention for BC patients	Literature review (multiple centers)	Systematic review	Systematic review and synthesis	No BC-specific mental health interventions found; identified critical gap in psychosocial support
Zhang [38]	Synthesize evidence on enhanced nursing care for self-efficacy and HRQoL in urostomy patients	Literature review (multiple centers)	Systematic review of 10 studies	Systematic review	Preoperative education critical for psychological preparation; postoperative interventions improved self-efficacy and HRQoL
Charalambous [20]	Scope trials of cancer nurse-led intervention across cancer care continuum	Multiple cancer centers globally	Scoping review of 214 studies	Scoping review	Most interventions during treatment phase; focused on education and counseling; improve multiple outcomes
Leow [39]	Develop and validate quality indicators for BC services	Multidisciplinary BC care centers	Multi-stakeholder collaboration	Guideline development	Established quality indicators for NMIBC/MIBC; emphasizes preoperative counseling, stoma marking ERAS protocols
Grassi [40]	Provide ESMO guideline on managing anxiety and depression in adult cancer patients	Guideline development consensus	Expert consensus	Clinical practice guideline (ESMO)	Recommend cognitive behavioral therapy and mindfulness; anxiety/depression common but underrecognized
Diefenbach [41]	Evaluate gratitude nursing program on fear of cancer recurrence in BC patients	Hosp oncology unit	*n* = 80 BC patients	Comparative study	Improved QoL, reduced fear, depression, anxiety; improved treatment compliance vs. routine care
Peng [23]	Assess people-oriented nursing mode on psychological status of BC patients	Hospital oncology department	*n* = 80 BC patients	Comparative study	Reduced anxiety and depression, improved QoL vs. conventional nursing
Kim [42]	Develop and test mobile-based mental health program for NMIBC patients	Ambulatory urology clinic	Pilot feasibility study	Protocol and feasibility	Mobile program via Kakao talk demonstrated feasibility; potential for mental health improvement
Leminski [43]	Evaluate combined educational and psychological support reducing perioperative anxiety in MIBC patients	Tertiary cancer center	*n* = 148 MIBC patients	Comparative study	Cystocare program significantly reduced perioperative depression (*p* < 0.001) and anxiety
Thomas [44]	Systematic review psychological distress and identify risk factors in BC patients	Literature review (multiple studies)	Systematic review of 17 studies (*n* = 2572)	Systematic review	Risk factors: advanced stage, younger age, female sex, preoperative; protective: social support
Wulff-Burchfield [45]	Qualitatively evaluate nurse-led preoperative stoma education for BC patients	Preoperative education clinic	*n* = 24 patients and caregivers	Qualitative evaluation	Interactive education with patients, advocates optimally prepares for ostomy and reduces distress
Zhang [46]	Narrative review of caregiver burden nursing education for BC patients with urinary diversion	Literature review (2018–2023)	Narrative review	Narrative literature review	Nurse-led stoma education enhanced caregiver comprehension and reduced burden/stress
Ding [47]	Evaluate peer-led education on stoma care and QoL in BC patients with permanent ostomy	Hospital stoma care clinic	*n* = 340 BC patients with ostomy	Randomized controlled trial	Peer-led intervention improved stoma care knowledge (*p* < 0.001) attitude, practices, and QoL
Solera-Gomez [48]	Scope educational needs for oncology nurses	Literature review (oncology settings)	Scoping review of multiple studies	Scoping review	Key needs: communication, coping, stress, prevention, continuous, technical skill development
Xu [49]	Synthesize best evidence for urinary incontinence management post-neobladder	Literature review	Evidence synthesis of multiple studies	Evidence synthesis	Comprehensive UI assessment, conservative, treatment, nursing equipment use, structured follow-up
Fan [24]	Evaluate internet plus health education on caregiver burden in COVID-19 era	Virtual and home-based platform	*n* = 80 caregivers of stoma patients	Randomized controlled trial	Internet plus education reduced caregiver burden and enhanced coping ability (*p* < 0.05)

BC: bladder cancer; COVID-19: Coronavirus Disease 2019; ERAS: enhanced recovery after surgery; ESMO: European Society of Medical Oncology; hosp: hospital; MIBC: muscle-invasive bladder cancer; *n*: number; *p*: probability; QoL: quality of life; RCT: randomized controlled trial; UI: urinary incontinence; and vs.: versus.

**Table 2:** Perioperative care enhanced recovery after (ERAS) protocols.

Author	Study Designs	Sample Size (*n*)	Primary Outcomes	Effect Size/Statistical Significance
Ashraf [37]	Retrospective comparative	94	Hospital stays reduction (11 vs. 17 days); complication reduction	35% LOS reductions; 38% complication reduction (*p* < 0.05)
Leminski [43]	Comparative study	148	Perioperative anxiety/depression reduction	Significant reduction in anxiety/depression scores (*p* < 0.001)
Leow [39]	Clinical guideline development	Experts’ consensus	Quality indicators establishment	Preoperative counseling, stoma marking, ERAS protocols standardized

LOS: length of stay; *p*: probability; vs.: versus; and ERAS: perioperative care and enhanced recovery after surgery.

**Table 3:** Intravesical therapy management and NMIBC outcomes with statistical evidence.

Author	Study Design	Sample Size	Main Findings	Effect Size/Statistical Significance
Wang [18]	Randomized controlled trial	100	Patients’ satisfaction compliance, self-efficacy, QoL improvement	Satisfaction (*p* < 0.001); compliance 100% vs. 84% (*p* < 0.05); anxiety reduction (*p* < 0.001)
Song [35]	Randomized controlled trial	126	Renal function preservation, reduced caregiver burden	Improved renal function, reduced burden/anxiety/depression (*p* < 0.05)

QoL = quality of life.

**Table 4:** Digital health and telehealth interventions with implementation data.

Authors	Intervention Type	Population (*n*)	Outcomes	Implementation Feasibility
Zhang [36]	Internet plus continuous nursing platform	Advanced BC with hematuria (43)	Improved coping, disease knowledge, reduced caregiver burden	Successfully implemented in hospital settings: scalable
Kim [42]	Mobile-based mental health (Kakao talk)	NMIBC patients’ pilot (*n* = feasibility)	Feasibility demonstrated; potential for mental health improvement	Pilot phase; ready for expansion
Diefenbach [41]	Web-based CRIS platform	BC survivor (7)	High usability; addresses practical/psychosocial/educational needs	User-friendly interface; practical information accessible
Fan [24]	Internet plus health education for caregivers	Caregivers of stoma patients (80)	Reduced caregiver burden; enhanced coping ability (*p* < 0.05)	Effective during COVID-19 pandemic; widely applicable

BC: bladder cancer; COVID-19: Coronavirus Disease 2019; NMIBC: non-muscle-invasive bladder cancer; *n*: number; and *p*: probability.

**Table 5:** Stoma and ostomy care management with evidence of effectiveness.

Author	Study Design	Sample Size (*n*)	Key Findings	Effect Size/Impact
Zhang [38]	Systematic review of 10 studies	10 studies reviewed	Preoperative education critical; postoperative care improved self-efficacy and HRQoL	Consistent improvement across studies; moderate to strong effect sizes
Wulff-Burchfield [45]	Qualitative evaluation	24 (patients and caregivers)	Interactive education optimally prepared patients; reduced distress	Qualitative evidence of psychological benefit and preparedness
Ding [47]	Randomized controlled trial	340	Peer-led education improved knowledge, attitudes, practices, QoL	Stoma knowledge (*p* < 0.001); sustained improvement
Zhang and Qi [46]	Narrative review	Literature 2018–2023	Enhanced caregiver comprehension; reduced burden/stress	Systematic evidence synthesis; reproducible outcomes.

QoL: quality of life; HRQoL: health-related quality of life.

## Data Availability

The original contributions presented in this study are included in the article/Supplementary Material. Further inquiries can be directed to the corresponding author.

## Supplementary Materials

The following supporting information can be downloaded at https://www.mdpi.com/article/10.3390/medicina62010185/s1, Table S1: PRISMA-ScR Checklist; Table S2: Complete Database Search Strings.

## Abbreviations

The following abbreviations are used in this manuscript:

BC	Bladder Cancer
CBT	Cognitive Behavioral Therapy
CI	Confidence Interval
COVID-19	Coronavirus Disease 2019
CRIS	Cancer Resource and Information System
ERAS	Enhanced Recovery After Surgery
ESMO	European Society of Medical Oncology
GSESs	General Self-Efficacy Scale Scores
Hosp	Hospital
HRQoL	Health-Related Quality of Life
LOS	Length of Stay
MIBC	Muscle-Invasive Bladder Cancer
*n*	Number
NMIBC	Non-Muscle Invasive Bladder Cancer
*p*	Probability (*p*-Value)
PRO	Patient Reported Outcomes
QoL	Quality of Life
RCT	Randomized Controlled trial
RR	Risk Ratio
SAS	Self-Rating Anxiety Scale
SDS	Self-Rating Depression Scale
UI	Urinary Incontinence

## References

[1] Zhang Y., Rumgay H., Li M., Yu H., Pan H., Ni J. (2023). The global landscape of bladder cancer incidence and mortality in 2020 and projections to 2040. J. Glob. Health.

[2] Saginala K., Barsouk A., Aluru J.S., Rawla P., Padala S.A., Barsouk A. (2020). Epidemiology of bladder cancer. Med. Sci..

[3] Abbas N.F., Aoude M.R., Kourie H.R., Al-Shamsi H.O. (2024). Uncovering the epidemiology of bladder cancer in the Arab world: A review of risk factors, molecular mechanisms, and clinical features. Asian J. Urol..

[4] Zuo J., Chen J., Tan Z., Zhu X., Wang H., Fu S., Wang J. (2025). Analysis of long-term trends and 15-year predictions of smoking-related bladder cancer burden in China across different age and sex groups from 1990 to 2021. Discov. Oncol..

[5] Kiebach J., Beeren I., Aben K.K.H., Witjes J.A., van der Heijden A.G., Kiemeney L.A.L.M., Vrieling A. (2025). Smoking behavior and the risks of tumor recurrence and progression in patients with non-muscle-invasive bladder cancer. Int. J. Cancer.

[6] Hussein A.A., Smith G., Guru K.A. (2023). The association between the urinary microbiome and bladder cancer. Urol. Clin. N. Am..

[7] Gherasim R.D., Chibelean C., Porav-Hodade D., Todea-Moga C., Tătaru S.O., Reman T.L., Vida A.O., Ghirca M.V., Ferro M., Martha O.K.I. (2025). Microbiome shifts in bladder cancer: A narrative review of urobiome composition, progression, and therapeutic impact. Medicina.

[8] Stamatakos P.V., Fragkoulis C., Zoidakis I., Ntoumas K., Kratiras Z., Mitsogiannis I., Dellis A. (2024). A review of urinary bladder microbiome in patients with bladder cancer and its implications in bladder pathogenesis. World J. Urol..

[9] Roje B., Zhang B., Mastrorilli E., Kovačić A., Sušak L., Ljubenkov I., Ćosić E., Vilović K., Meštrović A., Lozo Vukovac E. (2024). Gut microbiota carcinogen metabolism causes distal tissue tumours. Nature.

[10] Ginwala R., Bukavina L., Sindhani M., Nachman E., Peri S., Franklin J., Drevik J., Christianson S., Geynisman D.M., Kutikov A. (2025). Bladder cancer microbiome and its association with chemoresponse. Front. Oncol..

[11] Lucchesi C.A., Vasilatis D.M., Mudryj M., Ghosh P.M. (2023). Pesticides and bladder cancer: Mechanisms leading to anti-cancer drug chemoresistance and new chemosensitization strategies. Int. J. Mol. Sci..

[12] Williams S.B., Yapici H.O., Singhal P.K., Weimer I., Pathan F., Hyatt H.W., Lodaya K., Li H. (2025). Real-world economic burden and healthcare resource utilization of radical cystectomy and trimodal therapy for bladder cancer in the United States. Urology.

[13] Sylvester R.J., Van der Meijden A.P., Oosterlinck W., Witjes J.A., Bouffioux C., Denis L., Newling D.W.W., Kurth K. (2006). Predicting recurrence and progression in individual patients with stage Ta T1 bladder cancer using EORTC risk tables: A combined analysis of 2596 patients from seven EORTC trials. Eur. Urol..

[14] https://seer.cancer.gov/statistics-network/explorer/.

[15] Bahlburg H., Tully K.H., Bach P., Butea-Bocu M.C., Reike M., Roghmann F., Noldus J., Müller G. (2024). Improvements in urinary symptoms, health-related quality of life, and psychosocial distress in the early recovery period after radical cystectomy and urinary diversion in 842 German bladder cancer patients: Data from uro-oncological rehabilitation. World J. Urol..

[16] Brück K., Atema V., Leliveld A.M., Franckena M., Meijer R.P., van der Heijden M.S., Donders A.R., Witjes J.A., Uyl-de Groot C.A., van Hoogstraten L.M.C. (2025). Health-related quality of life in patients treated for nonmetastatic muscle-invasive bladder cancer: Radical cystectomy versus bladder-preserving therapy. Int. J. Radiat. Oncol. Biol. Phys..

[17] Cerruto M.A., D’Elia C., Siracusano S., Gedeshi X., Mariotto A., Iafrate M., Niero M., Lonardi C., Bassi P., Belgrano E. (2016). Systematic review and meta-analysis of non-RCTs on health-related quality of life after radical cystectomy using validated questionnaires: Better results with orthotopic neobladder versus ileal conduit. Eur. J. Surg. Oncol..

[18] Wang W., Chen Y., Gu J. (2024). Effectiveness of integrated nursing interventions in enhancing patient outcomes during postoperative intravesical instillation for non-muscle invasive bladder cancer: A comparative study. Medicine.

[19] Causey L., Leak Bryant A., Spencer Smith B., Coombs L.A. (2025). Incorporating nurse navigation to improve cancer survivorship care plan delivery. Clin. J. Oncol. Nurs..

[20] Charalambous A., Wells M., Campbell P., Torrens C., Östlund U., Oldenmenger W., Patiraki E., Sharp L., Nohavova I., Domenech Climent N. (2018). A scoping review of trials of interventions led or delivered by cancer nurses. Int. J. Nurs. Stud..

[21] Liu H., Yang K., Gong F., Wu Y., Tang S. (2021). Application of rapid rehabilitation nursing in perioperative period of laparoscopic radical prostatectomy for prostate cancer patients. J. Nanomater..

[22] Fearon K.C.H., Ljungqvist O., von Meyenfeldt M., Revhaug A., Dejong C.H.C., Lassen K., Nygren J., Hausel J., Soop M., Andersen J. (2005). Enhanced recovery after surgery: A consensus review of clinical care for patients undergoing colonic resection. Clin. Nutr..

[23] Peng F., Meng Y., Sun L., Dong B., Xu G., Liu S., Zhang X., Liu T. (2024). People-oriented nursing mode on the negative emotions and psychological status of patients with bladder cancer. Iran. J. Public Health.

[24] Fan X., Li H., Lai L., Zhou X., Ye X., Xiao H. (2022). Impact of internet plus health education on urinary stoma caregivers in coping with care burden and stress in the era of COVID-19. Front. Psychol..

[25] Han Y., Han Y., Huang W., Liu Y., Wang Z., Zhao W., Zhang W. (2025). Effects of nurse-led interventions on enhancing patient-related outcomes in colorectal cancer management throughout the cancer care continuum: A systematic review and meta-analysis. Int. J. Nurs. Stud..

[26] Hashemi N., Bahrami M., Tabesh E., Arbon P. (2022). Nurse’s roles in colorectal cancer prevention: A narrative review. J. Prev..

[27] D’Alessandro L., Morales Palomares S., Mancin S., Sguanci M., Cattani D., Cosmai S., Lopane D., Gjeloshi M., Cangelosi G., Mazzoleni B. (2025). Professional roles, skills and advanced educational programs of breast cancer nurses: A scoping review. Nurs. Open.

[28] Haugen M., Kelly K.P., Leonard M., Mills D., Sung L., Mowbray C., Landier W. (2015). Nurse-led programs to facilitate enrollment to Children’s Oncology Group cancer control trials. J. Pediatr. Oncol. Nurs..

[29] van Klinken M., Hafkamp E., Gualtherie van Weezel A., Rodin G., Schulz Quach C., de Vries F., Lo C., Hales S., Zimmermann C., Rydall A. (2023). Teaching oncology nurses a psychosocial intervention for advanced cancer: A mixed-methods feasibility study. Semin. Oncol. Nurs..

[30] Bessa A., Rammant E., Enting D., Bryan R.T., Shamim Khan M., Malde S., Nair R., Thurairaja R., Cahill F., Amery S. (2021). The need for supportive mental wellbeing interventions in bladder cancer patients: A systematic review of the literature. PLoS ONE.

[31] MacLennan S.J., MacLennan S. (2020). How do we meet the supportive care and information needs of those living with and beyond bladder cancer?. Front. Oncol..

[32] Shaffer K.M., Turner K.L., Siwik C., Gonzalez B.D., Upasani R., Glazer J.V., Ferguson R.J., Joshua C., Low C.A. (2023). Digital health and telehealth in cancer care: A scoping review of reviews. Lancet Digit. Health.

[33] Arksey H., O’Malley L. (2005). Scoping studies: Towards a methodological framework. Int. J. Soc. Res. Methodol..

[34] Tricco A.C., Lillie E., Zarin W., O’Brien K.K., Colquhoun H., Levac D., Moher D., Peters M.D.J., Horsley T., Weeks L. (2018). PRISMA Extension for Scoping Reviews (PRISMA-ScR): Checklist and explanation. Ann. Intern. Med..

[35] Song Y., Ren P., Wu Y., Zhang B., Wang J., Li Y. (2022). Efficacy of long-term extended nursing services combined with atezolizumab in patients with bladder cancer after endoscopic bladder resection. Medicine.

[36] Zhang M., Guo S., Gan S., Xu Q. (2024). “Internet Plus” continuous nursing for patients with advanced bladder cancer: A retrospective observational study. Medicine.

[37] Ashraf W., Hamid A., Malik S.A., Khawaja R., Para S.A., Wani M.S., Mehdi S. (2024). Integrated enhanced recovery after surgery protocol in radical cystectomy for bladder tumour—A retroprospective study. BJUI Compass.

[38] Zhang T., Qi X. (2023). Enhanced nursing care for improving the self-efficacy and health-related quality of life in patients with a urostomy. J. Multidiscip. Healthc..

[39] Leow J.J., Catto J.W., Efstathiou J.A., Gore J.L., Hussein A.A., Shariat S.F., Smith A.B., Weizer A.Z., Wirth M., Witjes J.A. (2020). Quality indicators for bladder cancer services: A collaborative review. Eur. Urol..

[40] Grassi L., Caruso R., Riba M., Lloyd-Williams M., Kissane D., Rodin G., McFarland D., Campos-Ródenas R., Zachariae R., Santini D. (2023). Anxiety and depression in adult cancer patients: ESMO Clinical Practice Guideline. ESMO Open.

[41] Diefenbach M.A., Marziliano A., Siembida E.J., Mistretta T., Pfister H., Yacoub A., Aibel K., Patel P., Lapitan E., Tagai E.K. (2023). Cancer Resource and Information Support (CRIS) for bladder cancer survivors and their caregivers: Development and usability testing study. JMIR Form. Res..

[42] Kim Y., Lee H., Park J., Lee S. (2023). A mobile-based mental health improvement program for non-muscle invasive bladder cancer patients: Program development and feasibility protocol. Eur. Psychiatry.

[43] Lemiński A., Kaczmarek K., Bańcarz A., Zakrzewska A., Małkiewicz B., Słojewski M. (2021). Educational and psychological support combined with minimally invasive surgical technique reduces perioperative depression and anxiety in patients with bladder cancer undergoing radical cystectomy. Int. J. Environ. Res. Public Health.

[44] Thomas K.R., Joshua C., Ibilibor C. (2024). Psychological distress in bladder cancer patients: A systematic review. Cancer Med..

[45] Wulff-Burchfield E.M., Potts M., Glavin K., Mirza M. (2021). A qualitative evaluation of a nurse-led pre-operative stoma education program for bladder cancer patients. Support. Care Cancer.

[46] Zhang T., Qi X. (2024). Caregiver burden in bladder cancer patients with urinary diversion post-radical cystectomy and the need for comprehensive nursing education: A narrative literature review. J. Multidiscip. Healthc..

[47] Ding J.-Y., Pan T.-T., Lu X.-J., You X.-M., Qi J.-X. (2024). Effects of peer-led education on knowledge, attitudes, practices of stoma care, and quality of life in bladder cancer patients after permanent ostomy. Front. Med..

[48] Solera-Gomez S., Benedito-Monleon A., Llinares-Insa L.I., Sancho-Cantus D., Navarro-Illana E. (2022). Educational needs in oncology nursing: A scoping review. Healthcare.

[49] Xu M., Chen S., Liu X., Luo Y., Wang D., Lu H., Jiang M., Chen X. (2025). Best evidence for rehabilitation management of urinary incontinence in patients with bladder cancer following orthotopic neobladder reconstruction. Asia-Pac. J. Oncol. Nurs..

[50] Biswas J., Bhuiyan A.K.M.M.R., Alam A., Chowdhury M.K. (2025). Effect of perceived social support on cancer patients: A narrative review. Acad. Oncol..

[51] Qian L., Zhang Y., Chen H., Pang Y., Wang C., Wang L., Zhang X. (2024). The clinical effect of gratitude extension-construction theory nursing program on bladder cancer patients with fear of cancer recurrence. Front. Oncol..

[52] Alqaisi O., Subih M., Joseph K., Yu E., Tai P. (2025). Oncology nurses’ attitudes, knowledge, and practices in providing sexuality care to cancer patients: A scoping review. Curr. Oncol..

[53] Alqaisi O., Al-Ghabeesh S., Tai P., Wong K., Joseph K., Yu E. (2025). A narrative review of nursing roles in addressing sexual dysfunction in oncology patients. Curr. Oncol..

[54] Horvath S., Carter N. (2024). Closing gaps in emergency care: The vital role of advanced practice nurses in serving vulnerable populations. Can. J. Emerg. Nurs..

[55] Bauernfeind L., Fels M., Dahlmann P., Rester C., Sterr F. (2024). The impact of advanced practitioners on patients in acute care—A mini review. Front. Disaster Emerg. Med..

[56] Bizzarri F.P., Campetella M., Recupero S.M., Bellavia F., D’Amico L., Rossi F., Gavi F., Filomena G.B., Russo P., Palermo G. (2025). Female sexual function after radical treatment for MIBC: A systematic review. J. Pers. Med..

